# Single Amino Acid Deletion at N-Terminus of the Target Antigen in DNA Vaccine Induces Altered CD8^+^ T Cell Responses against Tumor Antigen

**DOI:** 10.3390/vaccines9060540

**Published:** 2021-05-21

**Authors:** Takashi Imai

**Affiliations:** 1 Department of Microbiology and Immunology, Graduate School of Medical Sciences, Kyushu University, Fukuoka 812-8582, Japan; 0521773@gmail.com; 2 Department of Infectious Diseases and Host Defense, Graduate School of Medicine, Gunma University, Maebashi, Gunma 371-8511, Japan; 3 Department of Microbiology, Saitama Medical University, Moroyama-machi, Iruma-gun, Saitama 350-0495, Japan

**Keywords:** DNA vaccine, immunology, CD8^+^ T cell, antigen presentation, proteasome, tumor, cancer, N-end rule

## Abstract

Since CD8^+^ T cells have immunological memory and can eliminate tumor or infected cells, antigen-specific CD8^+^ T cell inducing DNA vaccines are potential next-generation vaccines. However, the relationship between single amino acid deletion of target antigens in plasmid DNA vaccines and vaccine efficacy is not completely understood. To address this knowledge disparity and improve DNA vaccine development, two constructs cytosolic form of ovalbumin, pOVAv (346 amino acids) and pOVAy (345 amino acids) were constructed and compared. OVA proteins from both constructs were detected in an in vitro experiment. Then, the efficacy of prophylactic DNA vaccination using a gene gun against OVA-expressing mouse thymoma cells was compared. Both constructs conferred protection against tumor challenge, and there was no significant difference between the efficacies of pOVAv and pOVAy. The pOVAv vaccine induced stronger antigen-specific cytotoxicity in vivo, while bone marrow-derived dendritic cells (BMDCs) transfected with pOVAv induced higher levels of IFN-γ production from OT-I CD8^+^ T cells in vitro compared to pOVAy. These results indicate that a single amino acid deletion at N-terminus of the target antigen in a DNA vaccine leads to a different immunological outcome. The small modification of the target antigen in the DNA vaccine might improve its efficacy against tumor or infectious diseases.

## 1. Introduction

Several types of vaccines, including live vaccines, attenuated vaccines, subunit vaccines, and DNA vaccines, are used to induce antigen-specific antibody production or T-cell responses [1]. A DNA vaccine is produced using plasmid DNA, and is thus relatively easy to prepare, low-cost, and has an excellent safety profile, as it does not require the use of the pathogen itself [2]. Additionally, DNA is more stable than proteins used for subunit vaccines, so it can be stored at room temperature and does not require refrigeration. Currently, multiple clinical trials evaluating the efficacy of DNA vaccines are ongoing. Some groups in the USA have been testing a DNA vaccine by Biojector^®^, VRC-HIVDNA016-00-VP, and have succeeded in potentiating an antibody and CD8^+^ T cell response against HIV [3,4]. Additionally, DNA vaccines against animal diseases, including West Nile virus of condors and horses “West Nile Innovator^®^ DNA” (Fort Dodge Animal Health/Pfizer, New York, NY, USA), infectious hematopoietic necrosis of salmonids“Apex-IHN^®^” (Aqua Health Ltd., an affiliate of Novartis Animal Health Inc., Wrentham, MA, USA), and canine melanoma “Oncept” (Merial, Lyon, France), are already commercially available [5,6,7,8].

CD8^+^ T cells are essential for eliminating tumors and intracellular pathogens, such as viruses, some types of bacteria, and parasites. CD8^+^ T cells recognize an antigen peptide, known as the epitope, and MHC class I complex on nucleated cells, and eliminate the cells in an antigen-specific manner. The antigen epitope is produced intracellularly through complex pathways, which include degradation by the ubiquitin-proteasome system, subsequent transfer to the ER for additional amino acid cleavage, and loading on empty MHC class I receptors for surface antigen presentation [9]. Thus, protein expression and degradation (antigen processing) are essential for producing epitopes for antigen presentation in a CD8^+^ T cell-inducing DNA vaccine strategy. 

During protein expression, the polymerase II type promoter initiates transcription from the template DNA [10]. In the DNA vaccine strategy, plasmid vectors pcDNA3.1, pCMV, pVIVO2, pCI, pVAX1, or pSV2 contain a cytomegalovirus or SV40 promoter, and these viral promoters drive protein expression more strongly than an endogenous promoter [11]. 

The first step in protein degradation is the covalent tagging of ubiquitin to a protein. Multiple ubiquitin-tagged proteins undergo proteasomal degradation [12]. In this ubiquitination process, specific proteins sequentially work as: (1) a ubiquitin-activating enzyme (E1), (2) a ubiquitin-conjugating enzyme (E2), and (3) a substrate-specific ubiquitin-protein ligase (E3) [13]. We previously reported that ubiquitin molecules fused with target proteins in pcDNA3.1 vector efficiently induce an antigen-specific killer CD8^+^ T cell response and potentiate DNA vaccines against tumors and infectious diseases [14,15,16,17]. Tobery and Siliciano also demonstrated that a recombinant viral vector expressing the ubiquitin-nef fusion construct induced cytotoxicity in vivo [18]. Another study reported that a ubiquitin-influenza virus nucleoprotein fusion DNA vaccine enhanced the antigen-specific CD8^+^ T cell response [19]. These ubiquitin-fusion strategies can skip the E1, E2, and E3 pathways as the rate-limiting stage in proteasomal degradation and are one way to potentiate DNA vaccines by inducing the CD8^+^ T cell response. 

The ubiquitin-proteasome system or autophagy plays important roles in the quality control of proteins and the recycling mechanism, which is responsible for degrading proteins into short peptides, or even at the amino acid level [20]. In the strategy to induce an antigen-specific CD8^+^ T cell response by DNA vaccine, both protein expression and degradation into the epitope seems to be essential. However, the relationship between a single amino acid deletion of the target protein in the plasmid construct of the DNA vaccine and the efficacy of the vaccine is not yet fully understood. In this study, to elucidate the molecular mechanism underlying DNA vaccine target-protein bioengineering, the second amino acid from the N-terminus of the model antigen ovalbumin (OVA) was constructed as either valine or deletion of valine. Then, the protein expression, the efficacy of DNA vaccine against tumors via gene gun, and induction of antigen-specific CD8^+^ T cell response were assessed.

## 2. Materials and Methods

### 2.1. Plasmid Construction and Isolation

**Figure 1:**
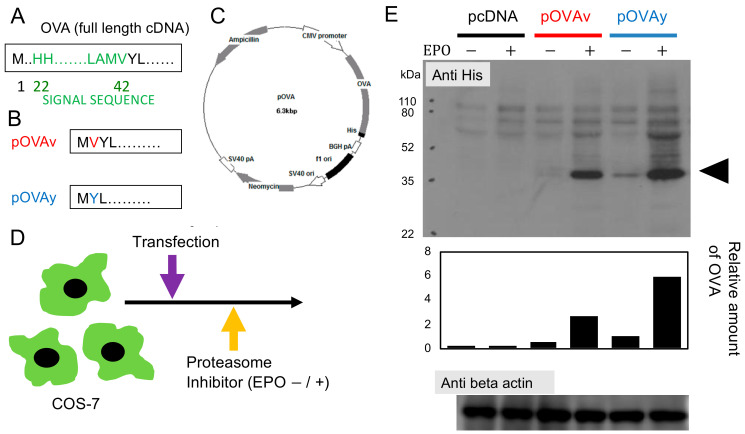
Plasmid construction and protein expression. (**A**) Ovalbumin (OVA) consists of 386 AA and has a signal sequence spanning from H22–V42. (**B**) The two plasmids used in this study start from the MV (pOVAv; 346AA) and MY (pOVAy; 345AA) of OVA without the signal sequence for the cytosolic form. (**C**) Plasmid map of pOVA (6.3 Kbp). Promoter: Cytomegalovirus (CMV), 6× His tag at the C-terminus for Western blotting. (**D**) COS-7 cells were transfected with plasmid pcDNA, pOVAv, or pOVAy and incubated with or without the proteasome inhibitor epoxomicin (EPO). (**E**) Western blotting for His tag of target proteins. β-actin was used as the internal loading control. The arrowhead indicates the expected cytosolic OVA size. The detected band of OVA was analyzed by ImageJ. A representative image from two independent experiments is shown.

The basic ovalbumin (OVA, from *Gallus gallus*) expressing plasmid pKEz–OVA [21] was kindly provided by Dr. Y. Itoh (Shiga University). The full length of OVA includes 386 amino acids, and the signal sequence starts from H22 to V42 (Figure 1A). The cytosolic form of OVA, in which most signal sequences were removed, was cloned into pcDNA3.1(-) (with cytomegalovirus promoter and 6× His tag at the C-terminus end) (Invitrogen, San Diego, CA, USA) between the 5′ *Xho*I and 3′ *Afl*II sites (Figure 1C). Two different plasmids, which started with either methionine valine (pOVAv, 346 amino acids (AA), 38.47 kDa) or methionine tyrosine (pOVAy, 345 AA, 38.37 kDa) [22], were produced (Figure 1B). The transformation was performed in competent DH5α cells, and the plasmid was isolated using plasmid mini- and maxi-prep kits (Sigma, St. Louis, MO, USA). Sanger sequencing was used to determine the sequence of the construct. 

### 2.2. Transfection and Western Blotting

COS-7 African green monkey kidney cells were cultured in 12-well tissue culture plates, and sub-confluent cells were transfected with 2 μg of plasmid DNA (pcDNA, pOVAv, pOVAy) using Lipofectamine 2000 (Invitrogen, San Diego, CA, USA). Cells were cultured for 48 h, and in the final 12 h, the proteasome inhibitor Epoxomicin (EPO) (Invivogen, San Diego, CA, USA) was added to half of the transfectants at a final concentration of 25 nM. 

Western blotting was performed by subjecting 40 μg of each cell lysate to SDS-PAGE, as previously described [15]. Anti-His (Sigma, St. Louis, MO, USA), anti-beta actin (Abcam, Cambridge, MA, USA), and peroxidase-conjugated anti-mouse IgG (H + L) (Zymed Laboratories, South San Francisco, CA, USA) were used as the primary and secondary antibodies, respectively. Protein detection was evaluated by chemiluminescence, and the protein expression level was analyzed using ImageJ (developed by Dr. Wayne Rasband (NIH)).

### 2.3. Mice and DNA Vaccination with a Gene Gun

C57BL/6 mice were purchased from Kyudo (Tosu, Saga, Japan) and SLC (Hamamatsu, Sizuoka, Japan). H-2K^b^-restricted, anti-OVA TCR transgenic mice (OT-I) [23] were kindly provided by Dr. Heath (Walter and Eliza Hall Institute, Australia). CD8^+^ T cells from OT-I mice recognize MHC class I (H-2K^b^-restricted) and the OVA epitope (SIINFEKL) complex. Female mice aged 8–14 weeks were used in this study. All experiments using mice were reviewed by the Committee for the Ethics on Animal Experiments in the Faculty of Medicine and conducted under the control of the Guidelines for Animal Experiments in the Faculty of Medicine, Kyushu University or Gunma University and the Law (No. 105) and Notification (No. 6) of the Government. 

**Figure 2:**
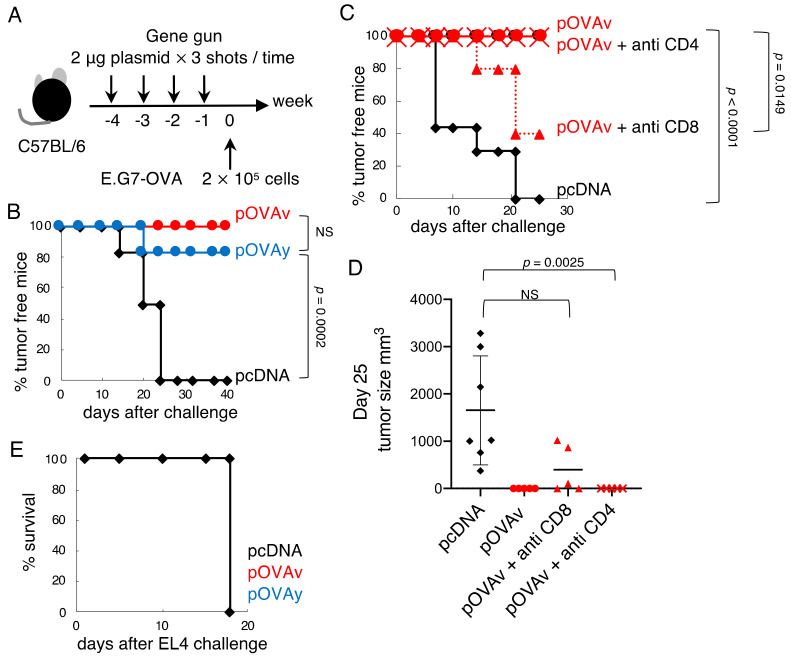
Anti-tumor effect of pOVA with four-time gene gun DNA immunization. (**A**) C57BL/6 mice were immunized four times with the DNA vaccine at one-week intervals and then challenged with E.G7-OVA tumor. (**B**) Percentage of tumor-free mice after immunization with pcDNA (black, *n* = 12), pOVAv (red, *n* = 8), pOVAy (blue, *n* = 12), and E.G7-OVA challenge. (**C**,**D**) Immunized mice were injected with anti-CD8, anti-CD4, or control antibodies before and after the E.G7-OVA challenge. The percentages of tumor-free mice are shown in (**C**). Tumor size at day 25 is shown in (**D**) (pcDNA [black]: *n* = 7, pOVAv [red circle]: *n* = 8, pOVAv + anti CD4 [red x]: *n* = 5, pOVAv + anti CD8 [red triangle]: *n* = 5). (**E**) Percentage survival after immunization and challenge with EL-4 tumor without OVA expression (*n* = 4/group). Data are the pooled results of one or two independent experiments.

**Figure 3:**
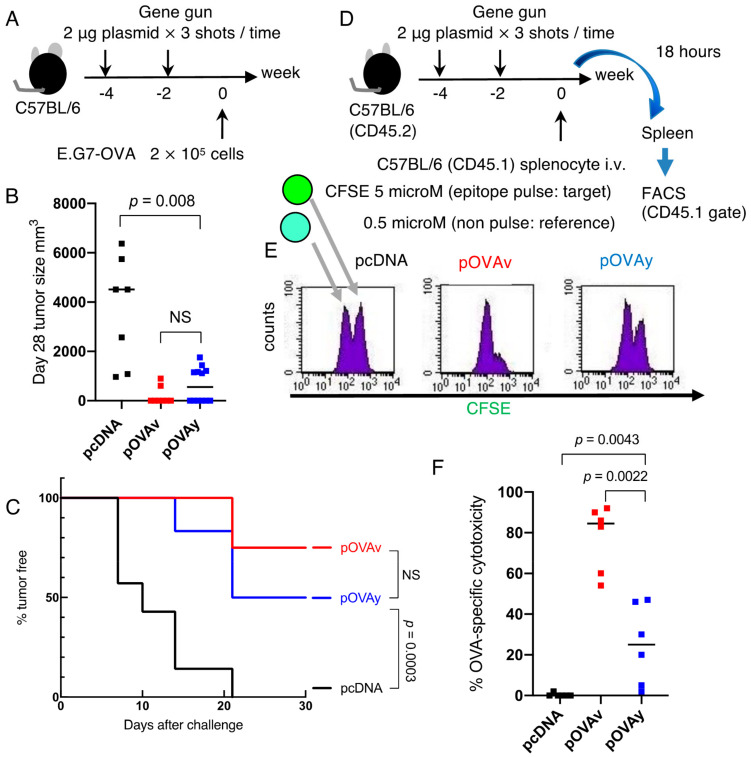
Comparison of pOVAv and pOVAy with two-time vaccine administration for anti-tumor immunity and OVA-specific cytotoxicity. (**A**) C57BL/6 mice were immunized twice with DNA vaccine via gene gun at two-week intervals. At week 0, the mice were challenged with E.G7-OVA. (**B**) Tumor size at day 28. Each symbol is indicative of an individual mouse. (**C**) Percentage of tumor-free mice with two-time immunization with pOVAv, pOVAy, or pcDNA. All data are pooled results of two independent experiments (pcDNA [black]: *n* = 7, pOVAv [red]: *n* = 8, pOVAy [blue]: *n* = 12). (**D**) In vivo cytotoxicity assay. C57BL/6 (CD45.2) mice were immunized 2 times with a gene gun same as A. On day 0, splenocytes from C57BL/6 (CD45.1) mice were separated into two groups, one group was OVA epitope (SIINFEKL)-pulsed and labeled with a high concentration of CFSE (5 μM; target cell for the vaccine), and the other group was epitope-non pulsed and labeled with a low concentration of CFSE (0.5 μM; reference cell of the assay). Both cells were mixed and transferred into C57BL/6 (CD45.2) mice. Then, the spleens were removed 18 h after transfer, and CD45.1 cells were stained with APC anti-CD45.1 and analyzed by FACS. CD45.1 cells were gated and analyzed based on the CFSE intensity (**E**). Left and right peaks include reference and target population, respectively. (**F**) Percent OVA-specific cytotoxicity. Each symbol is indicative of an individual mouse. Data are the pooled results of two independent experiments (*n* = 6/group).

We used a Helios gene gun system (Bio-Rad Laboratories, Hercules, CA, USA) to immunize mice, as previously described [15]. The mice were immunized four or two times on a shaved abdominal skin area with 6 μg of plasmid DNA (2-µg shot of plasmid DNA, 3 shots at once) using a gene gun at several intervals, as indicated in Figure 2A and Figure 3A,D.

### 2.4. Tumor Challenge, Depletion of T Cells, and In Vivo Cytotoxicity Assay

E.G7-OVA, an OVA-transfected thymoma cell line [24], or the mother cell line EL4 were injected subcutaneously on the back (2 × 10^5^ cells) several weeks after the final immunization (Figure 2: 1 week; Figure 3: 2 weeks), as indicated in the figures. Tumor size was measured twice weekly using calipers. 

To deplete the T cell subset, anti-CD4 mAb (clone GK1.5) or anti-CD8 mAb (clone 2.43) were injected intraperitoneally (0.5 mg) on days −1, +14, and +28 of the tumor challenge (day 0). Over 95% of the appropriate cell subset was depleted with this method, confirmed by flow cytometry using FACSCalibur (FACS) (Becton Dickinson, San Jose, CA, USA) and FlowJo (Treestar, Ashland, OR, USA). 

In vivo cytotoxicity assays were performed using carboxyfluorescein succinimidyl ester (CFSE; Molecular Probes, Eugene, OR, USA), as previously described [25]. Briefly, C57BL/6 (CD45.2) mice were immunized twice using a gene gun. On day 0, splenocytes from C57BL/6 (CD45.1) mice were separated into two groups. One group was H-2K^b^-restricted OVA epitope (SIINFEKL)-pulsed and labeled with a high concentration of CFSE (5 μM; target cell for the vaccine), while the other group was epitope-non-pulsed and labeled with a low concentration of CFSE (0.5 μM; reference cell of the assay). Both cells were mixed in a 1.1:1 ratio. CFSE at a high concentration has a tendency to damage the cells; thus, we used 10% more cells for the target than the reference. A total of 21 million cells were transferred into C57BL/6 (CD45.2) mice. The spleens were removed 18 h after transfer and CD45.1 cells were stained with APC anti-CD45.1 and analyzed by FACS. CD45.1 cells were gated and analyzed based on the CFSE intensity. 

### 2.5. BMDC Induction, Transfection, and OVA Antigen Presentation Assay In Vitro

Immature bone marrow-derived dendritic cells (BMDCs) from the bone marrow of C57BL/6 mice were induced by in vitro culture for 5 to 6 days in complete RPMI 1640 medium (supplemented with 2 mM L-glutamine, 1 mM sodium pyruvate, 0.1 mM nonessential amino acids, penicillin, streptomycin, and 2-ME) with 10% FCS and 20 ng/mL GM-CSF (R&D Systems, Minneapolis, MN, USA).

Immature BMDCs were transfected with 0.5 μg of plasmid DNA (pcDNA, pOVAv, pOVAy) using Nucleofector II and Mouse Dendritic Cell Nucleofector Kit with program Y-001 (Amaxa Biosystems, Cologne, Nordrhein-Westfalen, Germany). The cells were then cultured with or without EPO (at a final concentration of 1 μM) for 5 h, fixed with 0.5% paraformaldehyde/PBS for 10 min, placed in 1 M glycine/PBS for 15 min, washed with RPMI1640, and used as antigen-presenting cells (APCs). OT-I CD8^+^ T cells were isolated from the spleen by negative sorting using a CD8α^+^ T cell isolation kit (CD4, CD11b, CD11c, CD19, CD45R (B220), CD49b (DX5), CD105, Anti-MHC-class II, TER119, and TCRγδ) (Miltenyi Biotec, Bergisch Gladbach, Germany). APC (1 × 10^4^ cells) and OT-I CD8^+^ T cells (1 × 10^5^ cells) were cultured in a 96-well plate for 24 h. Then, culture supernatants were collected to measure IFN-γ. An ELISA kit for mouse IFN-γ (BioLegend, San Diego, CA, USA) was used in this study.

### 2.6. Statistical Analysis

Statistical evaluation of the differences between experimental groups was conducted using the Mann–Whitney U test. Statistical significance was set at *p* < 0.05. Significant differences in survival were tested with a log-rank test using Kaplan–Meier survival curves. GraphPad Prism (version 8.3.0; GraphPad Software, San Diego, CA, USA) was used for all statistical analyses.

## 3. Results

### 3.1. Expression of N-Terminus Modified Target Protein for DNA Vaccine

Plasmids carrying the cytosolic form of OVA as a model antigen were constructed. To compare the single amino acid deletion, the pOVAv (346AA; MVYL…) and pOVAy (345AA; MYL…) plasmid with one amino acid deletion at the 2nd residue from the N-terminal methionine (Figure 1B) were transfected into COS-7 cells. To confirm the proteasomal degradation of OVA, the proteasome inhibitor epoxomicin (EPO) was added to half of the culture. After harvesting the cells, Western blotting (anti-His antibody) was used to determine the expression of OVA (Figure 1D,E). β-Actin was used as an internal loading control. In COS-7 cells transfected with pOVAv and cultured without EPO, the lower band corresponding to cytosolic OVA was nearly undetectable. However, in pOVAv-transfected cells cultured with EPO, the cytosolic OVA band was clearly visible. In contrast, the OVA band was detected in pOVAy-transfected cells cultured without EPO, and the band intensity was further increased in pOVAy-transfected cells cultured with EPO. The OVA band in the pcDNA-transfected cells was not detected. These results demonstrate that the degradation of the OVA protein is dependent on the proteasome, which is important for MHC class I epitope production.

### 3.2. Anti-Tumor Effect of pOVAv and pOVAy with a Gene Gun

To elucidate the difference between the single amino acid deletion with regard to the induction of anti-tumor immunity, C57BL/6 mice were immunized four times via the administration of plasmid DNA with the gene gun, and E.G7-OVA cells (2.0 × 10^5^) were inoculated subcutaneously (Figure 2A). Control pcDNA did not have any anti-tumor effect, but both pOVAv and pOVAy strongly inhibited tumor growth. The pOVAv plasmid conferred complete resistance against the E.G7-OVA challenge, whereas pOVAy-inoculated mice were 85% tumor-free (Figure 2B); however, the difference was not statistically significant. This DNA vaccine against E.G7-OVA was dependent on CD8^+^ T cells, but not CD4^+^ T cells (Figure 2C,D), and was highly antigen-specific. Thus, no mice survived the challenge with EL4, the mother cell line of E.G7-OVA, which does not express OVA (Figure 2E).

Inoculating the mice four times with the DNA vaccine effectively suppressed E.G7-OVA. Next, vaccine administration was reduced from four to two times, and the anti-tumor effects of pOVAv versus pOVAy were compared (Figure 3A). There was no significant difference in tumor size at day 28 after E.G7-OVA inoculation between pOVAv and pOVAy with double DNA immunization, while the administration of the pOVA vaccine twice induced a stronger anti-tumor immunity than pcDNA (Figure 3B). Double immunization with pOVAv protected 75% of the mice from the E.G7-OVA challenge, but pOVAy protected only 50% of the mice (Figure 3C), with no significant difference between pOVAv and pOVAy. Reducing the number of DNA vaccinations weakened anti-tumor immunity. Taken together, these findings indicate that complete tumor rejection requires a four-time immunization. 

### 3.3. Cytotoxicity Induction by pOVAv Was Stronger Than pOVAy

Since the tumor challenge experiment was used to evaluate one-month host defense, the corresponding assay may not be able to evaluate small immunological differences between pOVAv and pOVAy. Thus, to evaluate short-term immunological outcomes after two-time gene gun vaccination, the target cells pulsed with OVA CTL epitope were mixed with epitope-non-pulsed reference cells and injected intravenously into vaccinated animals to determine the differences between the in vivo antigen-specific cytotoxicity of pOVAv and pOVAy. After 18 h, the inoculated cells were analyzed by FACS based on surface markers and the intensity of pre-labeling with CFSE (Figure 3D). In the pcDNA group, the target and reference cells were in a 1:1 ratio. In contrast, in the pOVAv vaccine group, the target population was much smaller than the reference group, and over 80% of the OVA epitope-pulsed cells were eliminated. Thus, compared to the pOVAy vaccine group, OVA-specific cytotoxicity was approximately 30% lower than that of pOVAv (Figure 3E,F; *p* = 0.0022). Taken together, these findings indicate that pOVAv was more effective than pOVAy in inducing antigen-specific cytotoxicity in the short term. 

### 3.4. Antigen Presentation by pOVAv Was Superior to pOVAy

**Figure 4:**
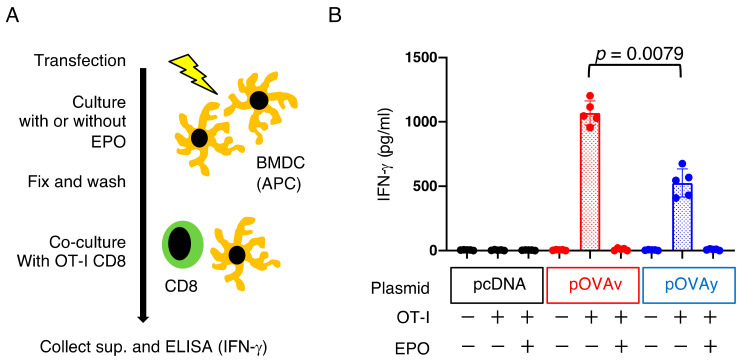
pOVAv induced higher levels of antigen presentation than pOVAy in vitro. (**A**) Protocol of endogenous antigen presentation assay. BMDCs were transfected with plasmid DNA and incubated for 5 h with or without proteasome inhibitor, and then fixed and co-cultured with OVA-specific CD8^+^ T cells from OT-I mice. The culture supernatants were collected to measure the levels of IFN-γ (see Materials and Methods). (**B**) IFN-γ production of the indicated experimental group. Data are representative of results from two independent experiments with similar results (*n* = 5/group).

CD8^+^ T cells must be primed by professional antigen-presenting cells (APCs), such as dendritic cells (DCs), prior to becoming effector or memory cells. In the final experiment, the efficiency of pOVAv and pOVAy as antigen-specific CD8^+^ T cell inducers was analyzed using a co-culture experiment with immature BMDCs as APCs and OT-I CD8^+^ T cells TCR-specific for MHC class I (H-2K^b^-restricted) and the OVA epitope (SIINFEKL) complex. Primed CD8^+^ T cells produce the cytokine IFN-γ, which is an indicator of antigen presentation [26] (Figure 4A). APCs transfected with pOVAv induced higher levels of IFN-γ production from OT-I CD8^+^ T cells than pOVAy (Figure 4B; *p* = 0.0079). Upon the addition of EPO to the culture, IFN-γ production was diminished in both pOVAv and pOVAy to the same level as the pcDNA-transfected APC group, demonstrating that this endogenously expressed OVA requires proteasomes for antigen presentation. These results indicate that pOVAv induced higher levels of IFN-γ production from OVA-specific CD8^+^ T cells than pOVAy in vitro.

## 4. Discussion

In this study, pOVAv constructs were found to generate a higher number or function of antigen-specific CD8^+^ T cells than pOVAy, indicating that single amino acid deletion result in different immunological outcomes. 

The regulation of protein stability and half-life by the amino acid at the N-terminus is referred to as the N-end rule [18]. In the present study, N-end amino acid deletion and/or variants were compared, and after transfection of COS-7 cells with either construct, treatment with the proteasome inhibitor EPO greatly enhanced the expression of OVA in both constructs (Figure 1). This suggests that both constructs are highly susceptible to proteasomal degradation. The difference in the detected OVA protein expression could be due to other factors. For example, it could be that the pOVAy construct has a better transfection rate or higher expression level, independent of proteasomal degradation. Thus, further studies are needed to fully elucidate the mechanisms underlying target protein expression and degradation. Researchers often struggle to express a given protein of interest [27]. Indeed, from our personal experience, a protein from a pathogen in a recombinant malaria parasite could not be detected. However, modification of the second amino acid or design the single amino acid deletion increased the protein expression level (Figure 1).

In general, the translated N-terminus amino acid of all proteins is methionine. However, this can be trimmed by posttranslational modifications [28]. According to Varshavsky [29,30], the pioneer of the N-end rule, the first amino acid in the Ac/N-end rule pathway, methionine, is cleaved from a specific second amino acid (Ala, Val, Ser, Thr, or Cys). Cleavage of N-end Met by Met-aminopeptidase often results in N-end-acetylation (Ac/N-end), which acts as a degradation signal (degron) [31]. Based on this report, Val would be N-end after cleavage of Met in pOVAv plasmid transfectant, while Tyr would be the second N-end (Met-Tyr-) in the pOVAy plasmid transfectant. If so the length of these OVA proteins would be the same in size. Ac/N-end Val from pOVAv might act as a degron, which might result in better antigen presentation and CD8^+^ T cell response than pOVAy. In addition to the N-end rule, Eisenlohr et al. proposed that hydrophobicity can be a driving factor in protein degradation and antigen presentation [32]. The presence of a Val residue may create a hydrophobic region that can destabilize a protein, leading to rapid degradation.

The relationship between the tertiary structures, stability, rate of proteolytic degradation, and antigen presentation of proteins is highly complex and is an interesting area of study [33]. In some instances, limited proteasomal degradation or protein stabilization leads to better antigen retention [34,35], favoring antigen presentation and T-cell activation. However, our studies in this area suggest the opposite. Moreover, there may be two routes to reach the target antigen via DCs, which are essential for priming CD8^+^ T cells by DNA vaccines [36]. In the case of the direct uptake of plasmid DNA by cutaneous DCs, Langerhans cells [37], protein translation occurs via episomes, where endogenous antigen processing produces epitopes in DCs [38]. The other route consists of plasmid DNA being introduced mechanically (e.g., helium gas pressure by gene gun) to non-professional antigen-presenting cells (e.g., epidermal cells, dermal cells, keratinocytes, and myocytes). Then, the translated target protein is released via apoptotic bodies or exosomes, followed by the phagocytosis of these vesicles by DCs, including the target protein, as well as the cross-presentation of the antigen by DCs [38,39]. In the latter case, stabilized proteins may maintain their antigenicity until DC uptake. On the other hand, with direct transfection to DCs, short-life proteins may induce a higher CD8^+^ T cell response due to the efficient production of epitopes for antigen presentation. 

We previously developed a ubiquitin fusion DNA vaccine that induces stronger cytotoxicity than normal DNA vaccines without ubiquitin fusion [14,15,16,17,22]. In this vaccine, the C-terminus amino acid of ubiquitin (the original C-terminus amino acid is G (glycine) at residue 76) is needed to stabilize the target antigen expression fused with ubiquitin. We previously demonstrated that mutated ubiquitin G76V fused with OVA (pUbV-OVAy) prevents the dissociation of ubiquitin from the OVA protein, which potentiates vaccine efficacy [22]. Thus, this is another example of the relationship between a single amino acid variant in a DNA vaccine and its immunological effects. In the present study, we investigated the target protein of a CD8^+^ T cell inducing DNA vaccine focusing on the N-end amino acid, since target protein expression and degradation are required for antigen presentation to CD8^+^ T cells. The proteasome inhibitor blocked OVA protein degradation (Figure 1E) and prevented antigen presentation (Figure 4B). Comparing the efficacy of the DNA vaccines in the present study (pOVAv and pOVAy) with those investigated in our previous study (pUbV-OVAy) [22], two-time pOVAv and pUbV-OVAy vaccines were found to have similar effects in terms of anti-tumor immunity and CD8^+^ T cell induction compared to the pOVAy vaccine, which was less effective (Figure 3). These data indicate that pOVAv confers the same level of immunity as ubiquitin fusion DNA vaccines. Nevertheless, pOVAv did not undergo ubiquitin fusion. Four-time immunization with pOVAv completely rejected the E.G7-OVA challenge (Figure 2B), suggesting that this regimen is effective in inducing sterile immunity.

## 5. Conclusions

Many studies are currently underway to potentiate the efficacy of DNA vaccines, including those investigating codon optimization, plasmid vector backbone optimization, molecular adjuvants, target protein expression enhancement, administration methods, and prime-boost strategies [11,39]. Some of these studies also investigated virus-like particle vaccines against papillomavirus [40] and subunit vaccines (protein vaccines) against influenza virus [41]. Target protein bioengineering provides useful insights for the development of better DNA vaccines, as well as ubiquitin fusion DNA vaccines. Our findings suggest that the combination of considering tiny difference in the size of target protein, N-end amino acid selection and ubiquitin fusion is a superior strategy for the development of a next-generation CD8^+^ T cell inducing DNA vaccine. 

## Data Availability

Data is contained within the article.
